# Proinflammatory Cytokines Enhance the Mineralization, Proliferation, and Metabolic Activity of Primary Human Osteoblast-like Cells

**DOI:** 10.3390/ijms252212358

**Published:** 2024-11-18

**Authors:** Juliana Franziska Bousch, Christoph Beyersdorf, Katharina Schultz, Joachim Windolf, Christoph Viktor Suschek, Uwe Maus

**Affiliations:** Department for Orthopedics and Trauma Surgery, Medical Faculty, University Hospital Düsseldorf, Heinrich Heine University Düsseldorf, 40225 Dusseldorf, Germany; julianafranziska.bousch@med.uni-duesseldorf.de (J.F.B.); christoph.beyersdorf@med.uni-duesseldorf.de (C.B.); katharina.schultz@med.uni-duesseldorf.de (K.S.); joachim.windolf@med.uni-duesseldorf.de (J.W.); suschek@hhu.de (C.V.S.)

**Keywords:** osteogenesis, osteoblasts, inflammation, proinflammatory cytokines, osteoporosis, proliferation, energy metabolism, osteoimmunology

## Abstract

Osteoporosis is a progressive metabolic bone disease characterized by decreased bone density and microarchitectural deterioration, leading to an increased risk of fracture, particularly in postmenopausal women and the elderly. Increasing evidence suggests that inflammatory processes play a key role in the pathogenesis of osteoporosis and are strongly associated with the activation of osteoclasts, the cells responsible for bone resorption. In the present study, we investigated, for the first time, the influence of proinflammatory cytokines on the osteogenic differentiation, proliferation, and metabolic activity of primary human osteoblast-like cells (OBs) derived from the femoral heads of elderly patients. We found that all the proinflammatory cytokines, IL-1β, TNF-α, IL-6, and IL-8, enhanced the extracellular matrix mineralization of OBs under differentiation-induced cell culture conditions. In the cases of IL-1β and TNF-α, increased mineralization was correlated with increased osteoblast proliferation. Additionally, IL-1β- and TNF-α-increased osteogenesis was accompanied by a rise in energy metabolism due to improved glycolysis or mitochondrial respiration. In conclusion, we show here, for the first time, that, in contrast to findings obtained with cell lines, mesenchymal stem cells, or animal models, human OBs obtained from patients exhibited significantly enhanced osteogenesis upon exposure to proinflammatory cytokines, probably in part via a mechanism involving enhanced cellular energy metabolism. This study significantly contributes to the field of osteoimmunology by examining a clinically relevant cell model that can help to develop treatments for inflammation-related metabolic bone diseases.

## 1. Introduction

Osteoporosis is a metabolic bone disease associated with low bone density due to enhanced bone tissue resorption or insufficient tissue formation, resulting in an increased risk of fracture. It has also been linked to the aging process and to chronic inflammation [1,2]. Chronic inflammatory diseases, such as rheumatoid arthritis and ankylosing spondylitis, can lead to reduced bone density and osteoporosis [3,4]. In metabolically active bone tissue, a continuous process called ‘bone remodeling’ occurs, in which old, micro-damaged bone is replaced by new, structurally stable bone tissue. Healthy bone requires a balance between the resorption of bone tissue by osteoclasts and the synthesis of new bone mass by osteoblasts [5]. A number of factors, including cytokines released by bone cells themselves, and especially during inflammation, can affect this balance. The interaction between bone metabolism and the immune system is a complex process summarized under the term osteoimmunology [6]. Cytokines can affect bone metabolism in a variety of ways, thereby disrupting the balance between bone resorption and formation. Inflammation is generally associated with increased bone resorption and metabolic bone diseases [7]. However, the aging process is also associated with chronic inflammation and can disrupt bone remodeling [8]. Mature osteoblasts, which differentiate from mesenchymal stem cells (MSCs), are required for the formation of new bone tissue. During this differentiation, known as osteoblastogenesis, cells secrete and mineralize an extracellular matrix (ECM) before differentiating into osteocytes [9]. Osteoblastogenesis is controlled by several signaling pathways, including Wnt, Notch, Hedgehog, bone morphogenic protein (BMP), and tumor growth factor-beta (TGF-β) [10]. Both proinflammatory and antiinflammatory cytokines can have diverse effects on osteoblastogenesis via different signaling pathways [11].

Proinflammatory cytokines, such as interleukin(IL)-1β and IL-6, as well as the chemokine IL-8 and tumor necrosis factor-alpha (TNF-α), are often described in the literature as promoters of bone resorption, but all show ambiguous roles [11,12]. IL-1β can induce osteoblast differentiation through the Wnt signaling pathway [13] and, conversely, inhibit it [14]. IL-6 triggers osteogenesis but also leads to the inhibition of osteoblastogenesis through downregulation of BMP signaling [11]. TNF-α inhibits the differentiation and proliferation of osteoblasts [11] but has also shown paradoxical results in several studies [15]. Similarly to IL-6, IL-8 is secreted by osteoblasts and is known to have a stimulating effect on the differentiation of osteoclasts [12]. However, IL-8 has also been observed to promote osteogenesis in vivo [16]. Taken together, these conflicting results reveal a complex and essential role of cytokines in bone metabolism. It should be noted that the majority of the studies cited summarize results from different cell models such as human MSCs or adipose stromal cells (ASCs), cell lines such as MC3T3-E1 or C2C12, or primary murine cells. There is a clear lack of results on the clinically relevant cell type, bone-forming osteoblasts from human bone tissue. In our study, we isolated primary human osteoblast-like cells (OBs) from the cancellous bone of human femoral heads to describe, for the first time, the influence of the proinflammatory cytokines IL-1β, IL-6, IL-8, and TNF-α during osteogenesis on the matrix mineralization, proliferation, and energy metabolism of these cells. This study should contribute to a better understanding of the activities of human bone cells under inflammatory conditions, as further research may lead to new therapeutic approaches for inflammation-related bone diseases, such as osteoporosis.

## 2. Results

**Figure 1:**
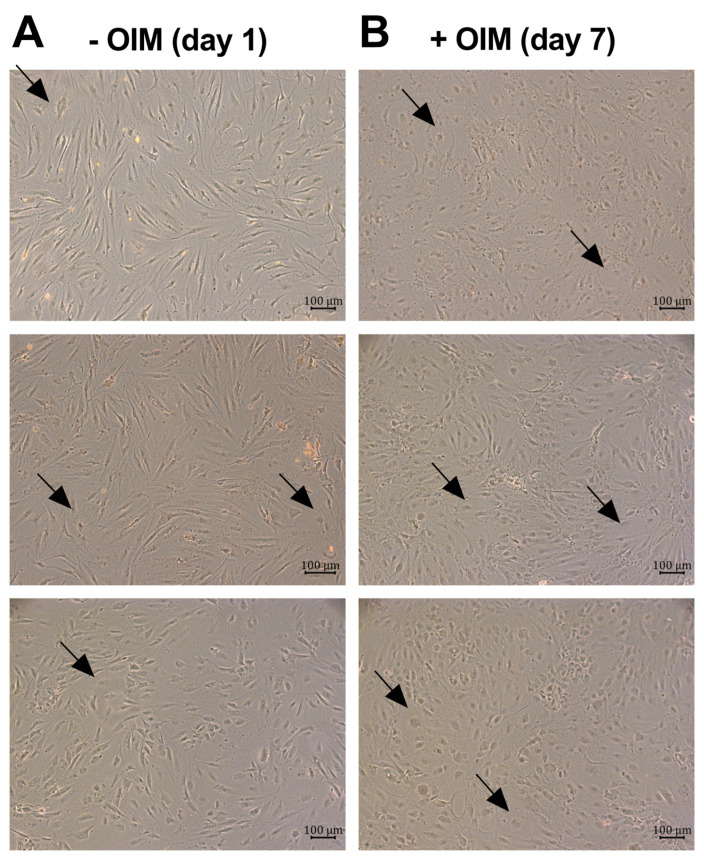
Morphologies of the osteoblast-like cell cultures. Light microscope pictures (100× magnification, scale bars = 100 µm) of OBs from three different donors seeded at confluency (**A**) on day 1 (undifferentiated cells) and (**B**) on day 7 of osteogenesis. The arrows mark roundish-shaped cells as an example.

First, the morphology of the osteoblast-like cells was observed with a light microscope. The undifferentiated confluent cells predominantly exhibited an elongated shape, although some cells had a more polygonal or roundish shape (Figure 1A). After seven days of osteogenesis, the cell morphology and arrangement in the culture changed notably (Figure 1B). Instead of the observed elongated shape, the cells became more rounded and formed regions of increased cellular density. The arrows in Figure 1 mark some roundish-shaped cells between the elongated-shaped cells as an example.

### 2.1. OBs Secreted the Proinflammatory Cytokines IL-6 and IL-8 During Osteogenesis

**Figure 2:**
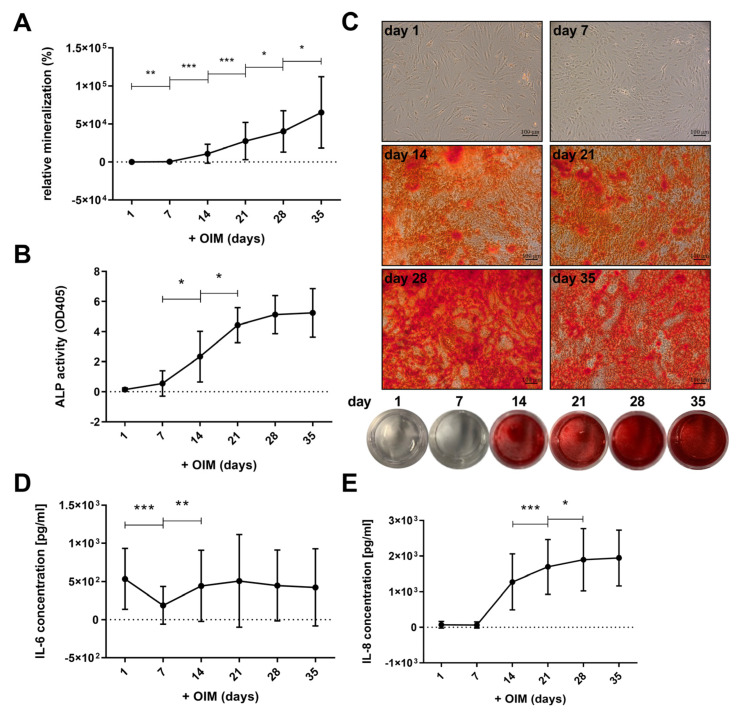
Interleukin (IL)-6 and IL-8 secretion during osteogenesis. The primary human osteoblast-like cells (OBs) were incubated in osteogenesis induction medium (OIM) for 35 days and (**A**) mineralization of the extracellular matrix (ECM) was quantified by Alizarin Red S staining (n = 13, four replicates each), (**C**) accompanied by example images of corresponding microscope images (100× magnification; scale bars = 100 µm) and well plates, and (**B**) alkaline phosphatase (ALP) activity was measured every week (n = 6, 4 replicates each). The protein concentration (pg/mL) of (**D**) IL-6 and (**E**) IL-8 in the supernatant was examined by enzyme-linked immunosorbent assay (ELISA) (n = 11, two replicates). Means with standard deviation (SD) are shown, and all significances were calculated by Wilcoxon signed-rank test (*p* ≤ 0.05 (*), *p* ≤ 0.01 (**), *p* ≤ 0.001 (***)).

Within the observed period of 35 days, the relative mineralization of ECM by primary human osteoblast-like cells (OBs) increased significantly during treatment with osteogenesis induction medium (OIM) (Figure 2A). Between days 7 and 14, the formation of calcified areas increased, as evidenced by microscopic and macroscopic observations (Figure 2C). The increase in mineralization was continuous until day 35. As shown in Figure 2B, the alkaline phosphatase (ALP) activity of the OBs also increased significantly between day 7 and day 21, and appeared to reach a plateau from this day onwards, until day 35.

During the osteogenesis, we observed a secretion of proinflammatory cytokines (Figure 2D,E). The measuring points represent values of cytokine secretion within one week during incubation in OIM. The undifferentiated cells secreted approximately 534 pg/mL of IL-6 (Figure 2D). A significant decrease in this secretion to about 187 pg/mL was observed on day 7. However, it increased again on day 14 to about the same level as the undifferentiated cells (day 1), with values between 423 and 507 pg/mL, and did not show significant changes until day 35. In contrast, IL-8 was barely secreted by undifferentiated OBs on day 1 (Figure 2E). From day 14 of osteogenesis, IL-8 secretion increased significantly to about 1275 pg/mL and continued to increase to 1947 pg/mL by day 35. No notable correlations were observed between the age or gender of the donors and the mineralization, ALP activity, or IL-6 and IL-8 secretion of the isolated cells (see Supplementary Figures S2 and S3).

### 2.2. The Proinflammatory Cytokines IL-1β, IL-6, IL-8, and TNF-α Increased the Mineralization of OBs

**Figure 3:**
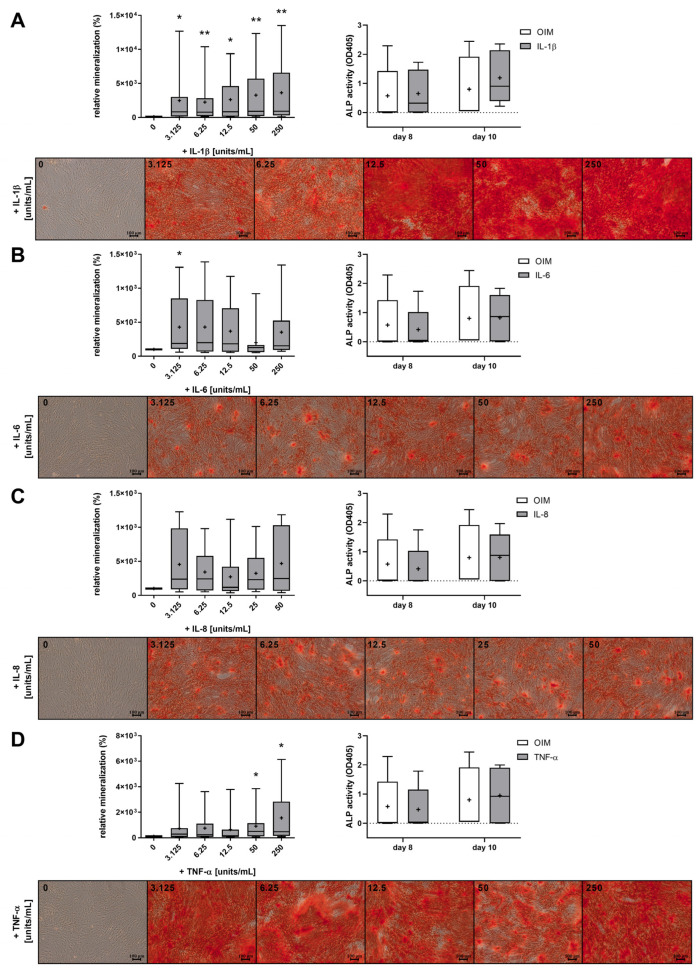
Effects of IL-1β, IL-6, IL-8, and tumor necrosis factor (TNF)-α on the mineralization of OBs. The boxplot diagrams illustrate the relative mineralization quantified by Alizarin Red S staining of OBs (n = 9, four replicates each) incubated for 21 days with the cytokines (**A**) IL-1β, (**B**) IL-6, (**C**) IL-8, and (**D**) TNF-α at concentrations ranging from 3.125 to 250 units/mL in OIM, with the corresponding microscopic images of the calcified nodules (scale bars ≙ 100 µm). Additionally, the ALP activity on day 8 and day 10 with selected concentrations of IL-1β (250 units/mL), IL-6 (12.5 units/mL), IL-8 (12.5 units/mL), and TNF-α (250 units/mL) is shown (n = 5, four replicates each). The whiskers representing the minimum and maximum, the median lines, and the means (+) are shown. The significances refer to the control (0 units/mL) and were calculated by the Wilcoxon signed-rank test (*p* ≤ 0.05 (*), *p* ≤ 0.01 (**)).

Treatment with IL-1β significantly induced the mineralization of the ECM by OBs at all concentrations (Figure 3A). The mineralization as a sign of the differentiation of OBs increasing proportionally with increasing concentrations of IL-1β and was already induced 24-fold at 3.125 units/mL IL-1β compared to the control (OIM without cytokines, 0 units/mL). At the highest concentration of 250 units/mL, the mineralization of the cytokine-treated OBs was 3648% stronger than that of the control cells. Treatment with IL-6 and IL-8 also resulted in a trend toward increased cell differentiation independent of the concentration, but at significantly lower values. The induction by IL-6 was about 356%, and that by IL-8 was about 373% higher compared to the control (Figure 3B,C). Only the lowest concentration of IL-6 led to a significant effect. The results with the TNF-α treatment were concentration-dependent, as with IL-1β, and with high concentrations of 50–250 units/mL, a significant induction of mineralization was measured, with up to 1552% higher values compared to the differentiated osteoblasts without cytokine treatment. The measurement of ALP activity as an early marker of osteogenic differentiation on day 8 and day 10 showed no significant increase, except for a slight trend in the IL-1β-treated cells on day 10. The whiskers and boxplots in Figure 3 reflect the natural variability inherent in the primary cell cultures. Despite this variability, some differences are statistically significant, underscoring the reliability of these results. No reductions in cell count were observed after 7 or 14 days with the cytokines of any tested concentration (Suppl. Figure S1), suggesting that the induction of apoptosis by the inflammatory factors is unlikely.

### 2.3. IL-6 and IL-8 mRNA Expression Were Enhanced by IL-1β and TNF-α

To understand the beneficial impact of cytokines on mineralization, we analyzed whether the cytokines influence each other’s reciprocal expression. Since we demonstrated the secretion of IL-6 and IL-8 during osteogenesis (Figure 2), we examined the influence of the cytokines on the mRNA expression of IL-6 and IL-8. The OBs were treated with the cytokines in OIM for 21 days. The highest concentrations (250 units/mL) were chosen for IL-1β and TNF-α, as these induced mineralization with the highest significant levels (Figure 3A,D). IL-6 and IL-8 were used at a concentration of 12.5 units/mL, as the effect on mineralization was independent of the concentration (Figure 3B,C).

**Figure 4:**
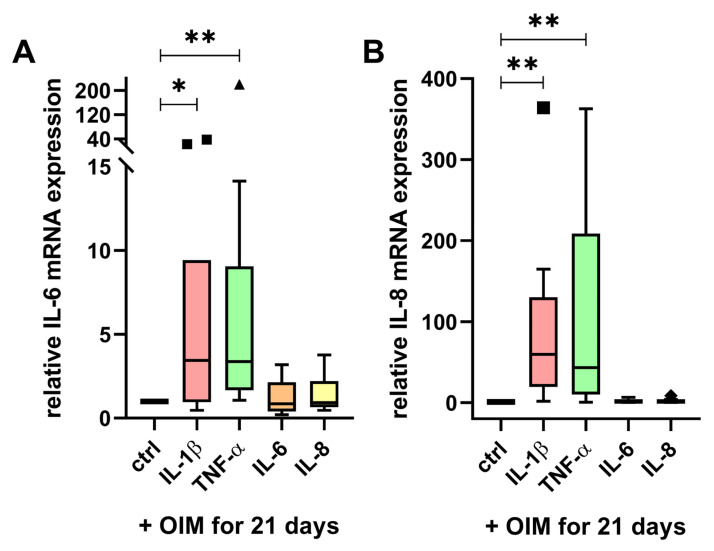
Reciprocal impact of the cytokines on mRNA expression in differentiated OBs. The mRNA expression of (**A**) IL-6 and (**B**) IL-8 was determined upon 21-day incubation of the cytokines IL-1β (250 units/mL), IL-6 (12.5 units/mL), IL-8 (12.5 units/mL), and TNF-α (250 units/mL) in OIM (n = 10, three replicates). The relative mRNA expression was calculated by the 2-ΔΔ Ct method and normalized on the control cells with 21-day OIM incubation without cytokines (ctrl) of each patient. The Tukey boxplot diagrams show the median line, and any values that exceed the threshold of the 75th percentile plus 1.5 times the interquartile range are plotted as statistical outliers (**■**, **▲**). The significances were calculated by the Wilcoxon signed-rank test (*p* ≤ 0.05 (*), *p* ≤ 0.01 (**)).

Treatment with IL-1β significantly increased the mRNA expression of IL-6 with an 8-fold higher mean value (Figure 4A) and the mRNA expression of IL-8 with a 91-fold higher mean value than the control (Figure 4B). Interestingly, the treatment with TNF-α resulted in an even stronger induction of IL-6 and IL-8. The mRNA expression of IL-6 was increased approximately 26-fold (Figure 4A), and the expression of IL-8 was increased about 104-fold by TNF-α (Figure 4B). Both IL-1β and TNF-α had a stronger impact on IL-8 than on IL-6 mRNA expression. The incubation with IL-6 and IL-8 did not affect their expression.

### 2.4. IL-1β and TNF-α Enhanced the Cell Count and Proliferation Rate of OBs During Osteogenesis

**Figure 5:**
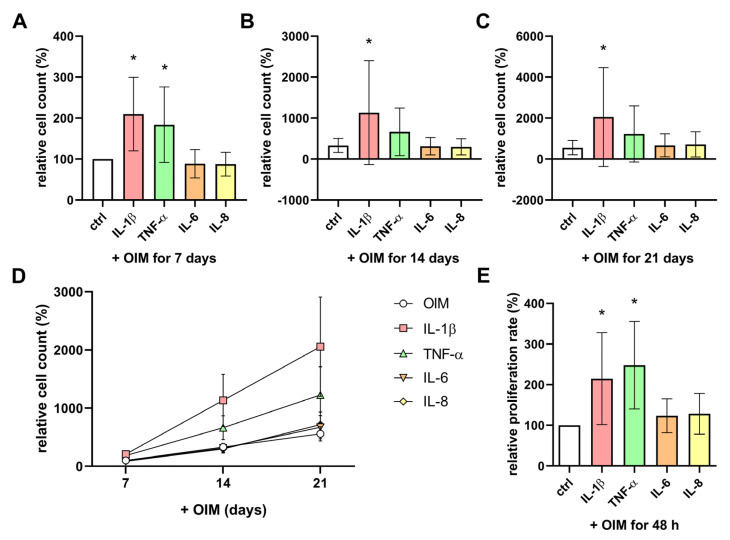
Impact of proinflammatory cytokines on the cell count and proliferation rate. The cell count of OBs incubated with the cytokines IL-1β (250 units/mL), IL-6 (12.5 units/mL), IL-8 (12.5 units/mL), and TNF-α (250 units/mL) in OIM on (**A**) day 7, (**B**) day 14, and (**C**) day 21 of osteogenesis was determined by Dimethylthiazolyl Blue Tetrazolium Bromide (MTT) assay. The mean with SD is shown. The diagram in (**D**) summarizes the observed period from day 7 to day 21 (n = 8, four replicates each); the mean with SEM is shown. All data were normalized for the control (OIM without cytokines) on day 7 from each patient. (**E**) The cell proliferation rate over 48 h was measured by bromodeoxyuridine (BrdU) assay (n = 6, four replicates each). The data were normalized for the control (OIM without cytokines) from each patient, and the mean with SD is shown. The significances were calculated by the Wilcoxon signed-rank test (*p* ≤ 0.05 (*)).

To analyze the influence of the cytokines on the cell count, the cells were incubated with or without cytokines in OIM for 21 days, and Dimethylthiazolyl Blue Tetrazolium Bromide (MTT) assays were performed. Already on day 7, the cell count of the OBs with IL-1β and TNF-α was significantly higher than the cell count of the control cells (Figure 5A). At day 14 and day 21, the effect of IL-1β remained significant with a cell count that was more than 3-fold higher compared to the control cells, but the cell count of the TNF-α-treated cells tended to be induced at both time points, albeit not significantly (Figure 5B,C). The cell counts of the OBs treated with IL-6 and IL-8 did not differ from the control during the observed period (Figure 5D). The examination of the proliferation rate using a bromodeoxyuridine (BrdU) assay confirmed the results of the MTT assay (Figure 5E). The OBs treated with IL-1β and TNF-α had significant, more than 2-fold higher, proliferation rates over a 48 h incubation period, while IL-6 and IL-8 had no effect. A comparison of the donors’ ages and the cell counts during osteogenesis with and without cytokines did not show a significant correlation (Suppl. Figure S4).

### 2.5. IL-1β and TNF-α Had Effects on Mitochondrial and Glycolytic Metabolism of the OBs

**Figure 6:**
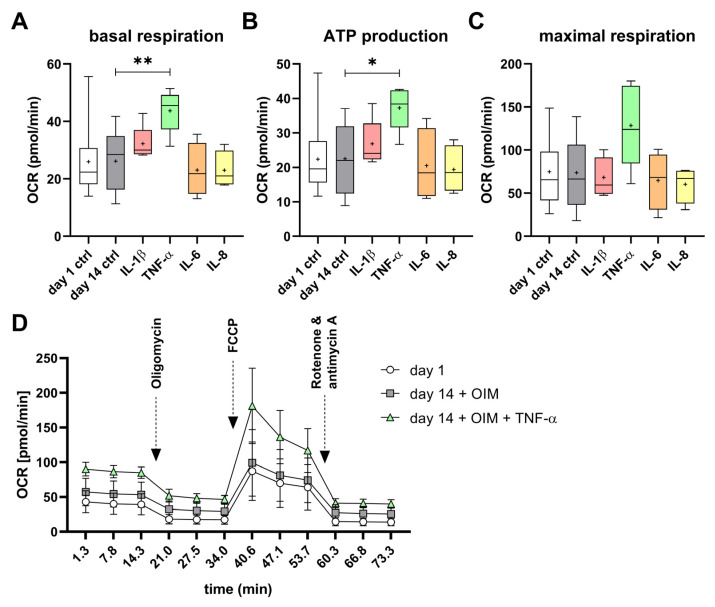
Impact of proinflammatory cytokines on the mitochondrial oxidative phosphorylation during osteogenesis. The (**A**) basal respiration, (**B**) adenosine triphosphate (ATP) production, and (**C**) maximal respiration were determined by Seahorse Cell Mito Stress Tests (Agilent). (**D**) All measurement time points of the assay for day 1, day 14 + OIM, and day 14 with OIM and TNF-α with the injection of assay reagents are shown. The OBs’ oxygen consumption rate (OCR) was measured on day 1 of osteogenesis (n = 16), and on day 14 without (n = 9) and with the cytokines IL-1β (250 units/mL; n = 5), IL-6 (12.5 units/mL; n = 4), IL-8 (12.5 units/mL; n = 4), and TNF-α (250 units/mL; n = 5). All experiments were performed with at least 10 technical replicates. The significances were calculated by the Mann–Whitney U test (*p* ≤ 0.05 (*), *p* ≤ 0.01 (**)).

No significant difference in basal respiration, adenosine triphosphate (ATP) production, or maximal respiration could be measured between the undifferentiated (day 1 control) and differentiated (day 14 ctrl) OBs (Figure 6A–C). Nevertheless, the oxygen consumption rate (OCR) of the differentiated OBs (day 14 + OIM) was slightly higher than that of undifferentiated OBs (day 1) throughout the assay (Figure 6D). Notably, treatment with TNF-α resulted in a significant 167% higher induction of basal respiration and significantly higher ATP production, of 166%, in the OBs compared to the day 14 control. However, there was no significant increase in maximum respiration. The treatments with the other cytokines, IL-1β, IL-6, and IL-8, did not result in significant changes (Figure 6A–C). The inductive effect of TNF-α on OCR was observed throughout the course of the assay (Figure 6D).

**Figure 7:**
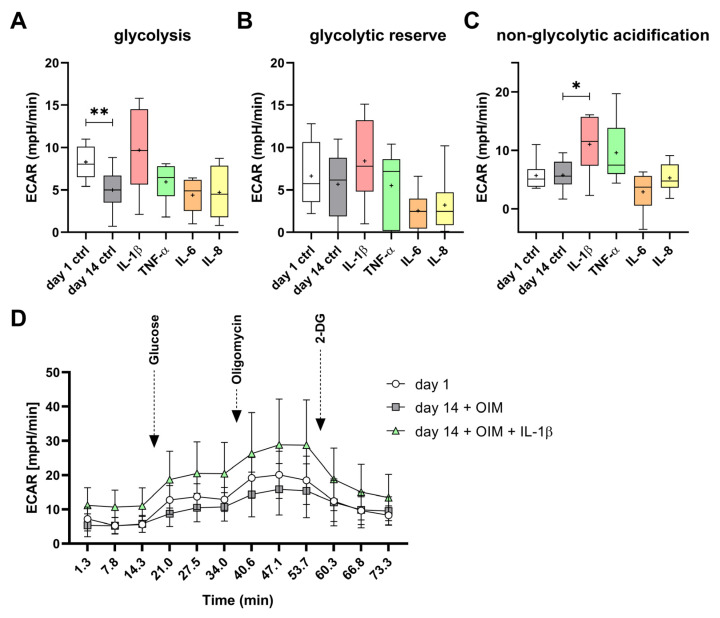
Impact of proinflammatory cytokines on glycolysis during osteogenesis. The (**A**) glycolysis, (**B**) glycolytic reserve, and (**C**) non-glycolytic acidification were determined by the Seahorse Glycolysis Stress Test (Agilent). (**D**) All measurement time points of the assay for day 1, day 14 + OIM, and day 14 with OIM and IL-1β with the injection of assay reagents are shown. The OBs’ extracellular acidification rate (ECAR) was measured on day 1 of osteogenesis (n = 8), and on day 14 without (n = 10, and with the cytokines IL-1β (250 units/mL; n = 6), IL-6 (12.5 units/mL; n = 6), IL-8 (12.5 units/mL; n = 6), and TNF-α (250 units/mL; n = 6). All experiments were performed with at least 10 technical replicates. The significances were calculated by the Mann–Whitney U test (*p* ≤ 0.05 (*), *p* ≤ 0.01 (**)).

The glycolysis of the differentiated OBs (day 14 ctrl) was significantly inhibited by about 60% compared to the undifferentiated OBs (day 1 ctrl). Interestingly, the cells treated with IL-1β showed a tendency to increase glycolysis and glycolytic reserve, although these changes were not statistically significant. The IL-1β-treated cells had a 194% higher glycolytic rate than the differentiated OBs without cytokines. TNF-α, IL-6, and IL-8 did not affect glycolysis compared to the day 14 control, but IL-6 and IL-8 tended to inhibit the glycolytic reserve (Figure 7A,B). This parameter defines the ability of the cell to respond to energy demand and is therefore a parameter of how close the glycolytic rate is to the theoretical maximum of the cell [17]. This is mimicked in the assay by the addition of oligomycin. The treatment with IL-1β induced significantly the non-glycolytic acidification, by 189%, compared to the day 14 control (Figure 7C). The increased basal extracellular acidification rate (ECAR) and the induced response to glucose of the IL-1β-treated OBs are shown in Figure 7D. Over the entire course of the assay, the ECAR of the IL-1β-treated cells was higher than those observed in the differentiated cells without cytokines and in the undifferentiated cells (day 1).

## 3. Discussion

In the present study, we demonstrate, for the first time, the effects of proinflammatory cytokines on mineralization, proliferation, and energy metabolism in primary human osteoblast-like cells as a cell model. We found that the cells secreted IL-6 and IL-8 during the process of osteogenesis and that treatment with IL-1β and TNF-α enhanced the mRNA expression of these cytokines. Interestingly, we observed that all the cytokine treatments for 21 days, especially IL-1β and TNF-α, led to increased osteogenesis. This was demonstrated by increased mineralization of the cells in the presence of ALP activity. At the same time, the cytokines had different effects on the proliferation and energy metabolism of the OBs.

It is known that cytokines significantly influence the differentiation of osteoclasts and osteoblasts. The cytokines TNF-α, IL-1β, IL-6, and IL-8 are generally considered to be osteoclastogenic, as they promote osteoclast differentiation and, thus, contribute to increased bone resorption [18]. However, previous studies on the effect of cytokines on osteoblast activity are ambivalent, and the impact likely depends on the concentration used and the cell model studied [11,12,15,16]. Although TNF-α is predominantly described as an inhibitor of osteogenesis [11], there are studies showing a positive effect on osteogenesis [15]. Similarly, IL-1β and IL-6 have been described as both positive and negative regulators of osteogenesis [11]. IL-8 is known to be produced by both osteoblasts and osteoclasts, but its effect on osteogenesis has not been well studied [12]. To contribute to a better understanding of these contradictory findings in different cell models, such as primary murine cells, cell lines such as MC3T3-E1 or C2C12, and human MSCs or ASCs, we tested the effect of cytokines on the more clinically relevant cell model, primary human OBs isolated from femoral heads of patients without osteoporosis, to investigate the baseline cellular behavior of cells from patients with healthy bone mass.

The observed IL-8 secretion during osteogenesis confirms findings with MSCs, in which IL-8 secretion also increased during differentiation into mature osteoblasts [19]. As observed in bone marrow mesenchymal stromal cells (BMSCs) and other cell types, we also confirmed the induction of IL-8 and IL-6 mRNA expression by TNF-α and IL-1β in OBs [20,21,22]. IL-8 seems to play an important role in osteogenesis and is significantly induced by other cytokines during inflammation. This could partly explain the observed increase in mineralization by TNF-α and IL-1β if they simultaneously induce other cytokines that promote mineralization, such as IL-8, albeit to a much lower extent. Thus, a combined cytokine effect could lead to enhanced cell differentiation, as observed with IL-1β and TNF-α. It would be interesting to study the combined effect of different proinflammatory cytokines to see whether an induction of mineralization would occur at an earlier stage. Interestingly, the increase in mineralization by the cytokine treatment did not correlate with an increase in ALP activity. It is conceivable that cytokine-induced mineralization occurs independently of ALP, or that it is indeed ALP-dependent but does not necessitate an increase in ALP activity to sustain this process. Ferreira et al. (2013) showed comparable results following the induction of the mineralization of human MSCs by IL-1β. Furthermore, the inhibition of ALP was shown to reverse the effect of IL-1β, leading to the conclusion that ALP is mandatory for matrix mineralization, but increased ALP activity is not necessary for the induction of enhanced mineralization [23]. Since we measured ALP activity simultaneously with mineralization, we assume enhanced osteogenesis of the cells.

Parallel to autocrine effects, IL-8 secretion during osteogenesis could also have paracrine functions in bone cell communication, as it has been described as an osteoclastogenic cytokine [20]. In addition to osteoclasts, osteal macrophages (osteomacs) also play an important role in the bone remodeling process and regulate osteoblast function [24]. Macrophages also have high IL-8 receptor expression, and their proinflammatory cytokine production is enhanced by IL-8 [25]. While we observed a positive effect of cytokines on osteogenesis in an osteoblast culture, the induction of osteoclastogenesis could be mediated by the osteoblasts themselves through IL-8 secretion in vivo, particularly in an inflammatory environment with TNF-α and IL-1β. In future studies, it will be important to investigate whether the increased activity of osteoclasts induced by cytokines impairs the induction of osteoblast activity, as observed here, and whether bone resorption occurs at a faster rate than bone synthesis. The use of osteoblast and osteoclast cocultures could be used to further investigate the communication between bone cells under inflammatory conditions [26].

During differentiation, downregulation of OB proliferation would be expected, with mature osteoblasts secreting the ECM and subsequently initiating mineralization [9,11]. However, our findings show that IL-1β and TNF-α significantly induce both cell proliferation and mineralization. Despite generally negative effects on osteogenic differentiation, these cytokines have previously been shown to have positive effects on the proliferation of osteogenic lineage cells [27,28,29]. The observed increase in proliferation rate due to IL-1β and TNF-α treatment is a possible explanation for the stronger induced mineralization compared to IL-6 and IL-8 treatment, as the higher cell count on day 21 could contribute to this effect.

Due to hormonal differences in donor sex and physical changes with age, we analyzed whether there were differences in osteogenesis or cytokine expression in OB cultures. When analyzing the correlation between age and the observed results, the cells of the middle age group (60–70 years) showed a decreased rate of mineralization during late osteogenesis compared to the oldest group (>70 years), while the other age groups (<60 years and >70 years) showed no statistically significant differences (Suppl. Figure S2). However, there was no effect on ALP activity. In addition, the cells from the middle age group had the lowest levels of IL-6 and IL-8 secretion, albeit not to a significant degree, and showed the least influence of cytokines on cell count during osteogenesis (Suppl. Figures S3 and S4). To confirm these observations, it would be necessary to include more donors in the study, thus increasing the size of the groups. The same applies to the comparison of donor sex, in which a slight but non-significant tendency towards higher mineralization and ALP activity in cells from female donors compared to male donors, as well as higher IL-6 and IL-8 secretion during osteogenesis, was observed (Suppl. Figures S2 and S3).

During osteogenesis and the formation and mineralization of the ECM, osteoblasts have an increased energy demand, which is provided at various stages of osteogenic differentiation through different metabolic pathways. Therefore, metabolic changes occur during osteogenesis, primarily involving oxidative phosphorylation (OXPHOS), glycolysis, or the tricarboxylic acid (TCA) cycle [30,31]. These changes depend on the cell model studied and the timing of measurements during differentiation, with multiple switches between the major energy sources likely occurring throughout this process. Undifferentiated MSCs prefer glycolysis and increase the rate of oxygen consumption by activated OXPHOS after 14 days of differentiation, whereas in murine calvarial osteoblasts, both pathways increase during early differentiation [32,33]. In the murine osteoblast cell line MC3T3-E1, an increase in glycolysis was observed at day 21 of differentiation, while OXPHOS was decreased compared to undifferentiated cells [34]. The present study did not detect any changes in OXPHOS at day 14 of differentiation in the OBs, but glycolysis was significantly inhibited compared to the undifferentiated control. Furthermore, treatment with IL-1β and TNF-α, both significant inducers of osteogenesis in OBs, also altered energy metabolism by increasing the ATP demand of the cells. The significant increase in OXPHOS by TNF-α suggests a slightly more mature differentiation phase compared to control cells not treated with cytokines. A significant increase in OXPHOS could also lead to the generation of excessive reactive oxygen species, possibly mitigated by a subsequent switch to glycolysis during the late matrix mineralization phase [33]. IL-1β, on the other hand, tended to increase glycolysis and significantly increased non-glycolytic acidification, which could indicate the use of the TCA cycle or glycogenesis [35]. This may suggest an even later differentiation phase of OBs, aligning with the increased mineralization observed with IL-1β treatment.

The partially divergent effects of these cytokines on osteoblast ATP production may activate distinct signaling pathways, despite the fact that they all lead to increased cell differentiation. A key signaling pathway involved is the p38 MAPK pathway, which mediates the increase in IL-6 production in osteoblasts and chondrocytes in response to IL-1β and TNF-α [36]. In human MSCs, it has also been shown that IL-1β can induce their osteogenic differentiation after 10 days via the Wnt-5a/Ror2 signaling pathway, but it can also induce specific miRNA, which in turn leads to the repression of β-catenin expression, thus inhibiting Wnt-driven osteogenesis [13,14]. These are just a few examples of cytokine-induced signaling pathways that target osteogenic differentiation. Future studies should prioritize the investigation of the underlying signaling pathways that regulate mineralization under inflammatory conditions. This may clarify the discrepancies observed in the current research and facilitate a deeper understanding of the mechanisms underlying inflammatory bone diseases such as osteoporosis. This study shows that energy metabolism and increased ATP production may play an important role in cytokine signaling and osteogenesis.

The differences in the effects of IL-1β, TNF-α, and IL-6 on the OBs’ differentiation could be relevant regarding anti-cytokine therapies, which are commonly used in the treatment of chronic inflammatory diseases such as RA. IL-1β, TNF-α, and IL-6 have stimulatory effects on osteoclast differentiation and bone resorption [18]. This observation aligns with the effectiveness of anti-TNF-α and anti-IL-6 therapies. Monotherapy with tocilizumab, an antibody against the IL-6 receptor, as well as an antibody against IL-1Ra, has been shown to inhibit the progression of structural joint damage in RA patients [37]. Similarly, treatment with anti-TNF-α (infliximab) in RA patients led to the arrest of spine and hip bone loss with a decrease in bone resorption markers like RANKL [38]. Additionally, Infliximab has also been shown to inhibit IL-1β and IL-6 gene expression in the human osteosarcoma cell line MG-63 [39]. Based on the findings in this study, blocking cytokines, particularly IL-1β and TNF-α, may not only inhibit osteoclastogenesis, but could also potentially limit osteoblast-driven matrix mineralization. To address this, combination therapies with bone anabolic agents that promote bone formation may be beneficial, reducing bone resorption without inhibiting bone formation.

It is acknowledged that this study is not without limitations. Measuring energy metabolism or mRNA expression at specific time points during osteogenesis might miss important changes in cells. Although working with human primary cells is valuable, these cells are derived from patients who have suffered a fracture or had coxarthrosis prior to isolation, implying an inflammatory preload and increasing the risk of sampling bias. It should be noted that osteoarthritis (OA) has a significant impact on bone health. Increased expression of cytokines in chondrocytes, along with the degradation of cartilage matrix proteins and other factors, also has an influential impact on bone cells [40]. This could potentially affect the results obtained with primary cells from OA patients, possibly leading to the observed variability between donor cells. It is also noteworthy that among the OA donors, there were a few cell cultures with a low response to cytokines on ECM mineralization. However, due to the relatively small number of donors, it was not possible to identify a pattern that could be attributed to donor OA status. The variability observed could have been due to individual differences between the patients’ medical histories, which requires further investigation. Moreover, it is important to recognize that OA and fractures involve disparate inflammatory responses, with OA being marked by a chronic low-grade inflammatory process, whereas fractures trigger an acute inflammatory reaction that reaches its peak within the initial 24 h following the injury [41,42]. This temporal and qualitative difference in inflammation may lead to phenotypic and functional differences in cells derived from these patients. However, due to relatively small sample sizes and inherent variability between patients, including age and medication use, it remains challenging to investigate the effects of chronic versus acute inflammation on human osteoblast function. To minimize the potential for bias related to pathological bone conditions, patients with normal bone density, as determined by DXA, indicating a normal balance of bone remodeling, were selected for inclusion in this study. Further studies comparing osteoblasts from OA and fracture patients would be necessary to better understand the specific impact of different inflammatory conditions. Individual variations among patients due to previous illnesses, age, and medication intake can also occur and cannot be avoided with a relatively small patient sample size. Despite using an established isolation method for primary osteoblast cultures, contamination by other bone cells and their precursors, such as osteomacs, cannot be entirely ruled out and could influence osteoblast activity [24]. To maintain a pure cell population, only low-passage cells were used. Finally, it is important to note that these were in vitro studies with 2D cell cultures. In vivo, cells interact with a matrix and other cells that are not present in cell culture. They are also exposed to mechanical loading in vivo, which can alter their response to cytokines. It was shown that IL-1 coupled with mechanical load caused less ECM degradation of bovine articular cartilage than IL-1 alone [43]. The nitric oxide release induced by IL-1β in primary chondrocytes was also suppressed by additional mechanical loading [44]. Thus, the results observed here cannot be directly transferred to 3D systems, and follow-up studies are required. Although the cells were treated with cytokines for 21 days, this period may not be sufficient to mimic the chronic inflammation seen in patients with osteoporosis. Future studies should compare OBs from the non-osteoporotic patients studied here with cells from osteoporotic patients exposed to a chronic inflammatory environment.

The partly contradictory results in the literature show the complexity of the regulation of bone remodeling and the necessary communication of bone cells, and probably depend on the cell model investigated and the experimental conditions. While most studies report negative effects of proinflammatory cytokines on hMSCs’ differentiation to osteoblasts, our results show that cytokines can promote the osteogenesis of mature osteoblasts under the selected conditions. Primary human OBs provide a realistic and clinically relevant model to study the effects of cytokines on bone metabolism. This study improves our understanding of the complex interplay between inflammation and the development of osteoporosis. Further research in this area could pave the way for new therapeutic strategies to prevent and treat inflammation-related metabolic bone diseases.

## 4. Materials and Methods

### 4.1. Materials

The cell culture materials were obtained from Sarstedt AG & Co. KG (Nümbrecht, Germany). Unless otherwise specified, all other materials and reagents were obtained from Merck KGaA (Darmstadt, Germany).

### 4.2. Bone Material, Ethics Approval, and Patient Information

The isolation and use of primary human osteoblast-like cells (OBs) was approved by the local Research Ethics Committee of the Heinrich Heine University Düsseldorf (Study No. 5585R). The patients had given written consent and had undergone arthroplasty due to osteoarthritis or fracture at the Clinic for Orthopedic and Trauma Surgery at the University Hospital of Düsseldorf (Germany). The bone density of each patient’s spine (L1–L4) and proximal femur was quantified by dual-energy X-ray absorptiometry (DXA). Femoral heads from patients with t-scores > −2.5 (non-osteoporotic) [45], acquired during surgery, were included in this study and stored in phosphate-buffered saline (PBS) with 1% penicillin/streptomycin (Pen/Strep) by PAN-Biotech GmbH (Aidenbach, Germany). A total of 28 patients (20 female, 8 male) with a mean age of 66 years (SD = 12.4) and a range of 31 to 88 years were included in this study. A detailed list of age, sex, condition, medications, and experiments in which the donor cells were used is provided in the Supplementary Materials (Supplementary Table S1).

### 4.3. Isolation of Human Osteoblast-like Cells

In order to isolate the OBs from the femoral heads, the cancellous bone was scraped out. The bone pieces were thoroughly rinsed with Dulbecco’s Modified Eagle Medium (DMEM), high-glucose (Gibco^®^ by Life Technologies^TM^, Carlsbad, CA, USA), and 1% Pen/Strep. They were then placed in a Falcon tube with 2.5 mg/mL collagenase type IV (Gibco^®^ by Life Technologies^TM^, Carlsbad, CA, USA), dissolved in OB growth medium. The bone pieces were digested for 2.5 h at 37 °C and the supernatant containing the cells was transferred to a new Falcon tube. After centrifugation at 400× *g* for 5 min, the supernatant was discarded and the cells were resuspended in OB growth medium and seeded into a T75 cell culture flask [46,47].

### 4.4. Cell Culture

The OB growth medium was composed of DMEM, high-glucose, with 10% fetal bovine serum (FBS), 1% Pen/Step, and 1% 4-(2-hydroxyethyl)-1-piperazineethanesulfonic acid (HEPES) solution. The osteogenesis induction medium (OIM) consisted of DMEM, high-glucose, 10% FBS, and 1% Pen/Strep, with the addition of 50 µM L-ascorbic acid 2-phosphate, 10 mM β-glycerophosphate disodium salt hydrate, and 500 nM dexamethasone [47,48]. The recombinant human cytokines IL-1β (1 unit/mL = 1 pg/mL), IL-6 (1 unit/mL = 100 pg/mL), IL-8 (1 unit/mL = 10 ng/mL), and TNF-α (1 unit/mL = 50 pg/mL) were obtained from Peprotech (Hamburg, Germany). The cells were maintained in a humidified chamber at 37 °C with 5% CO_2_.

### 4.5. Alizarin Red S Staining

The Alizarin Red S staining method was employed to quantify the calcium phosphates present in the mineralized ECM [49]. In 24- or 48-well plates, OBs were seeded at confluency and were treated with OIM for up to 35 days and with cytokines for 21 days to induce differentiation. For the Alizarin Red S stain, the cells were fixed with 4% formaldehyde for 15 min, followed by two washes with PBS. Subsequently, the cells were incubated with the 2% Alizarin Red S mono sodium salt staining solution for 20 min at 37 °C. Following several washes with deionized water, images of the wells were captured. The stain was redissolved with 10% (1-Hexadecyl)pyridinium chloride monohydrate (CPC; Thermo Fisher Scientific GmbH, Karlsruhe, Germany) for approximately 1 h in a shaker. The absorption of the dye, indicative of the mineralized ECM, was determined photometrically (OD600).

### 4.6. Extracellular ALP Activity

To determine the extracellular activity of alkaline phosphatase (ALP), the OBs were seeded in confluency in 48-well plates. The cells were treated with OIM for a period of up to 35 days or with cytokines for 10 days. To remove any residual medium, the cells were washed with PBS. This was followed by the addition of 750 µL of the ALP substrate p-nitrophenyl phosphate, and the plates were incubated at 37 °C for 10 min. The substrate’s conversion to yellowish p-nitrophenol by active ALP was determined in a photometer at a wavelength of 405 nm [50].

### 4.7. Quantification of Cytokines in Cell Supernatants by Enzyme-Linked Immunosorbent Assay (ELISA)

For the quantification of secreted cytokines, the OBs were seeded in 6-well plates. Upon reaching confluency, the medium was first replaced with OB growth medium, which was collected after 6 days for the ‘day 1’ sample. This was followed by starting the induction of the OB differentiation by incubation in OIM for up to 35 days. The supernatant containing the secreted proteins was collected every week and replaced with fresh OIM. The cytokine concentrations were determined by photometric measurement using the sandwich ELISA DuoSet^®^ Human IL-6 ELISA and DuoSet^®^ Human IL-8/CXCL8 ELISA with the DuoSet^®^ Ancillary Reagent Kit 2 by R&D Systems (Minneapolis, MN, USA). The tests were performed according to the manufacturer’s instructions.

### 4.8. Real-Time qPCR

In 6-well plates, the OBs were seeded at confluency and incubated in OIM with and without cytokines for 21 days. Total cell RNA was isolated after lysing the cells using the RNeasy^®^ Mini Kits (QIAGEN N.V., Hilden, Germany). The RNA was converted into cDNA using the Omniscript^®^ RT Kits (QIAGEN N.V., Hilden, Germany). RT-qPCR was performed in triplicates using the Power SYBR^®^ Green PCR Master Mix (Thermo Fisher Scientific GmbH, Karlsruhe, Germany) with 10 ng cDNA for IL-6 and 1 ng cDNA for IL-8 in a 25 µL PCR reaction with a 7300 Real-Time PCR System (Applied Biosystems, Waltham, MA, USA). For the determination of mRNA expression, primer concentrations of 0.2 µM for IL-6 and 0.4 µM for IL-8 were used. Transferrin receptor 1 (TFRC) was used as the reference gene for normalization. The forward and reverse primer sequences are listed in Table 1. The 2-ΔΔ Ct method was employed for the calculation of relative mRNA expression [51].

### 4.9. MTT Assay for Cell Count Quantification

Dimethylthiazolyl Blue Tetrazolium Bromide (MTT) assays were performed to quantify cell count, with a starting cell number of 7.5 × 10^2^ OBs per well in 96-well plates. After 24 h, the cells were treated with and without the cytokines in OIM for up to 21 days. On days 1, 7, 14, and 21, the yellowish MTT was added to the cells in growth medium (0.5 mg/mL) and incubated at 37 °C for 2 h. Viable cells converted the MTT into purple formazan, which was redissolved with dimethyl sulfoxide (DMSO) [52]. The absorbance was measured photometrically at 540 nm.

### 4.10. BrdU Assay for Cell Proliferation Rate

The proliferation rate of the cells was examined using the colorimetric bromodeoxyuridine (BrdU) kit, manufactured by Roche Holding AG (Basel, Switzerland). In total, 2 × 10^4^ OBs per well were seeded in a 48-well plate. After 48 h of resting time, the cells were treated with OIM in the presence or absence of cytokines with the addition of 10 µM BrdU labeling solution. Following a 48 h incubation period with the cells at 37 °C, the cells were fixed by incubation in FixDenat^TM^ for 30 min. Subsequently, the anti-BrdU-POD working solution was incubated for 90 min. After 15 min of TMB substrate incubation, its conversion was stopped by adding 1 M sulphuric acid, followed by the measurement in the photometer at 450 nm.

### 4.11. Seahorse Assay: Evaluation of Oxygen Consumption Rate (OCR) and Extracellular Acidification Rate (ECAR)

For the metabolic assays, all materials were purchased from Agilent Technologies, Inc. (Santa Clara, CA, USA), and the tests were performed according to the manufacturer’s instructions, unless otherwise noted. In total, 1.5 × 10^4^ OBs per well were seeded in a 96-well cell culture microplate and after 24 h, the treatment with and without cytokines in OIM was started for 14 days. Oxidative phosphorylation (OXPHOS) was quantified using the Seahorse XF Cell Mito Stress Test Kit (CMST), and glycolysis was quantified using the Seahorse XF Glycolysis Stress Test Kit (GST). Before measurement, cells were incubated in Seahorse XF Base Medium with 2 mM L-glutamine (Gibco^®^ by Life Technologies, Carlsbad, CA, USA) for 30–60 min without CO_2_ at 37 °C. The following concentrations of the reagents were used: 1 µM oligomycin, 1 µM carbonyl cyanide-p-trifluoromethoxyphenyl-hydrazone (FCCP), and 1 µM rotenone/antimycin A for the CMST, and 10 mM glucose, 1 µM oligomycin, and 50 mM 2 deoxy-D-glucose (2-DG) for the GST. The oxygen consumption rate (OCR) and the extracellular acidification rate (ECAR) were measured using a Seahorse XFe96 analyzer, and Seahorse Wave Desktop Software (Version 2.6.3) by Agilent Technologies, Inc. (Santa Clara, CA, USA) was used to evaluate the data. After the measurement, the cells were stained for 5 min with 1:1000 diluted Hoechst, and the fluorescence intensity was measured (excitation: 361 nm, emission: 486 nm) to normalize the data to the cell count.

### 4.12. Microscopic Images

The microscopic images in Figure 1 were taken with the BZ-X810 microscope by Keyence Corporation (Osaka, Japan), and the images in Figure 2 and Figure 3 were taken with the Axiovert 2000 microscope by Carl Zeiss AG (Oberkochen, Germany) at 100× magnification.

### 4.13. Statistics

The statistical analyses were performed using the GraphPad PRISM 8 software (Boston, MA, USA). The data were treated as non-parametric. Therefore, the Wilcoxon signed-rank test was used for the statistical analysis of paired data, and the Mann–Whitney U test was used for unpaired data. *p*-values lower than 0.05 were considered statistically significant. Details on the respective biological and technical replicates performed are provided in the corresponding figure caption.

## Figures and Tables

**Table 1:** List of RT-qPCR Primers.

Target Gene	Forward Primer (5′->3′)	Reverse Primer (5′->3′)	Product Length
IL-6	TCCAGTTCCTGCAGAAAAAGGCAA	TGGTTCTGTGCCTGCAGCTT	100
IL-8	CCACCGGAAGGAACCATCTCAC	CCTTGGCAAAACTGCACCTTCAC	114
TFRC	TTCAGGTCAAAGACAGCGCTCA	CTATACGCCACATAACCCCCAGG	100

## Data Availability

The raw data supporting the conclusions of this article will be made available by the authors on request.

## Supplementary Materials

The following supporting information can be downloaded at: https://www.mdpi.com/article/10.3390/ijms252212358/s1.
