# Supplementary material for: Proinflammatory Cytokines Enhance the Mineralization, Proliferation, and Metabolic Activity of Primary Human Osteoblast-like Cells
Source: Int J Mol Sci. 2024 Nov 18;25(22):12358. doi: 10.3390/ijms252212358 (PMC11594863; doi:10.3390/ijms252212358)
Supplement: Supplementary file 1 [file ijms-25-12358-s001.zip › ijms-3232929-supplementary.pdf]

Supplements

List of Tables:

Supplementary Table S1: Information about the donors involved in the study, including age, sex, condition, medications, and the list of the experiments in which the donor cells were used.

List of Figures:

Supplementary Figure S1: Influence of the applied concentrations of the cytokines (A) IL-1β, (B) IL-6, (C) IL-8, and (D) TNF-α (see Figure 2) on the cell count (n = 3), determined by MTT assay.

Supplementary Figure S2: Influence of donor age and sex on mineralization and ALP activity of osteoblast-like cells *in vitro*.

Supplementary Figure S3: Influence of donor age and sex on IL-6 and IL-8 secretion of osteoblast-like cells *in vitro*.

Supplementary Figure S4: Influence of donor age on the cell count during osteogenesis with and without cytokine treatment of osteoblast-like cells *in vitro*.

Supplementary Table S1: Information about the donors involved in the study, including age, sex, condition, medications, and the list of the experiments in which the donor cells were used.

	age	sex	condition	medication	experiments
1	70	f	coxarthrosis	Cotrimoxazol, Carvedilol, Pantoprazol, Torasemid, Digitoxin, Xipamid, ASS, Prednisolon, Trospiumchlorid, Sevelamer, Levemir	A, C, D, S
2	83	f	coxarthrosis	Allopurinol, Ferrosanol, Estradiol, Lansoprazol, Metformin, Doxepin, Gabapentin, Circadin, Atorvastatin, Folsäure, Torasemid, Doxazosin, Eliquis	A, C, D, H
3	78	f	coxarthrosis	ASS, Atorvastatin, Metoprolol, Ramipril, HVT, Lercanidipin, Pantoprazol	A, D, H
4	77	f	fracture	Amiodaron, Candecor, Nebivolol, Nexium, L-Thyroxin, Simvastatin, Spiolto, Eliquis, Torasemid	A. H
5	54	f	coxarthrosis	ASS, Omeprazol, Zanipress	A, C, D, E, H
6	56	m	coxarthrosis		A, B, C, E, H
7	88	m	fracture	Marcumar, Ferrosanol, Allopurinol, Amlodipin, Xipamid, Bicanorm	A, C, D, E, H
8	70	m	coxarthrosis		A, C, D, E, H
9	78	m	fracture	L-Thyroxin, Ramipril, Atorvastatin, Metohexal, Dostinex, Pantoprazol, Mirtazapin, Ergenyl Chrono, Amlodipin, Hydrocortison, Tamsublock, Duo, Spasmex, Macrogol, Restex	A, E
10	77	f	coxarthrosis	Lercanidipin, Metohexal, Opipramol, Simvahexal, Candesartan, Torem	A, D, E, F, G, H, I
11	55	f	coxarthrosis	Amlodipin, Doxepin, Pantozol, Lisihexal	A, C, E
12	60	f	coxarthrosis		A, B, C, F, G, H, I, J
13	79	m	fracture	Amiodaron, Atorvastatin, Metoprolol, Apixaban, ASS, Torasemid, Spironolacton, Foster Spray, Tamsulosin, Allopurinol, Zopiclon, EPO	A, B, D, F, H, I
14	66	f	coxarthrosis	Ramipril, Amlodipin, Triamtorem/HCT	B, F
15	57	f	coxarthrosis		B, G, H, J
16	57	f	coxarthrosis		C, E, H
17	67	m	coxarthrosis	Enalapril	C, D, H, S
18	50	f	coxarthrosis	Pantoprazol, Venlaflaxin, Ramipril, Metoprolol, HCT, L-Thyroxin, Hydromophon	C, F, G, H, I
19	64	m	coxarthrosis		E
20	77	f	fracture	Amiodaron, Candecor, Nebivolol, Nexium, L-Thyroxin, Simvastatin, Spiolto, Eliquis, Torasemid	E
21	69	f	coxarthrosis	Pantoprazol, L-Thyroxin, Valsartan, Spasmex	F, H, I
22	31	f	coxarthrosis		G, H, I, J
23	55	f	coxarthrosis	Amlodipin, Doxepin, Pantozol, Lisihexal	B, F, I
24	77	f	fracture	Marcumar, ASS, Amiodaron, Bisoprolol, Pantozol, Rosuvastatin, Ezetimib, Azopt	F, G, H, I
25	63	m	fracture	Xarelto, ASS, Metoprolol, Ramipril, Torem, Simvastatin, Citalopram, Mirtazaptin, Foster Spray	I

26	53	f	coxarthrosis	ASS, Ramipril, Pantozol, Torasemid, Spironolacton, Metoprolol, Digitoxin, Lercanidipin, Atorvastatin	I
27	65	f	coxarthrosis	N.A.	J
28	81	f	coxarthrosis	N.A.	J
Only included in supplemental results (Fig. 2)					
29	97	f	coxarthrosis	Amlodipin, Folsan, Novalgin, Ramipril, Tilidin, Torasemid, Zopiclon, Paracetamol, Tinzaparin	S

Experiment legend:

A mineralization (Fig. 2 A)

B ALP activity (Fig. 2 B)

C IL-6/IL-8 secretion (Fig. 2 D/E)

D mineralization with cytokines (Fig. 3)

E IL-6/IL-8 mRNA expression (Fig. 4)

F cell count with cytokines during osteogenesis (Fig. 5 A-D)

G proliferation rate with cytokines (Fig. 5 E)

H oxidative phosphorylation (Fig. 6)

I glycolysis (Fig. 7)

J ALP activity with cytokines (Fig. 3)

S cell count with cytokine concentrations in Supplements (Suppl. Fig. 1)

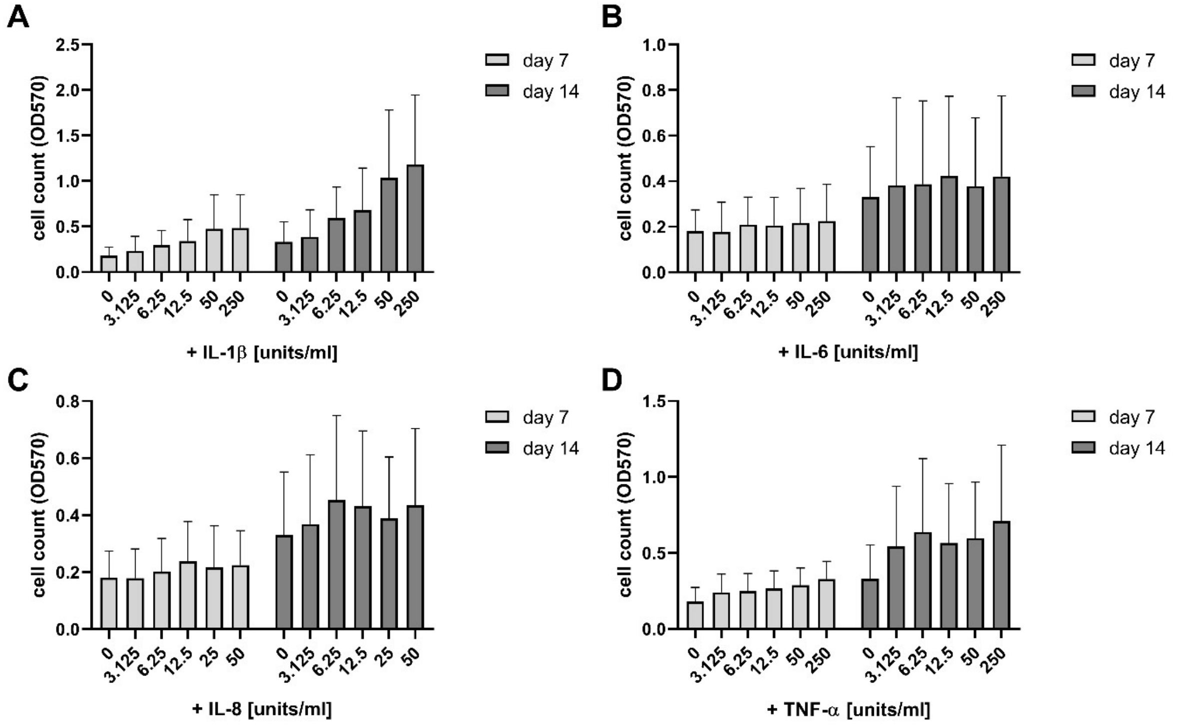

Supplementary Figure S1: Influence of the applied concentrations of the cytokines (A) IL-1β, (B) IL-6, (C) IL-8, and (D) TNF-*α* (see Figure 2) on the cell count (n = 3), determined by MTT assay.

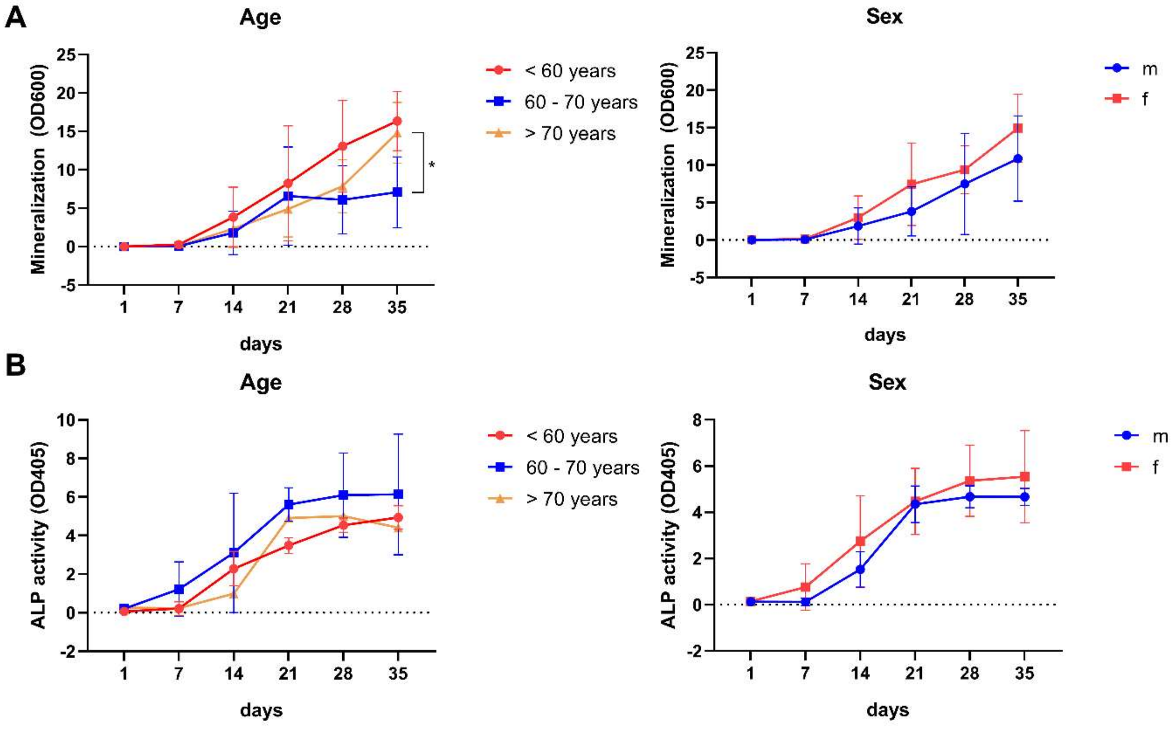

Supplementary Figure S2: Influence of donor age and sex on mineralization and ALP activity of osteoblast-like cells *in vitro*. (A) The results of Fig. 2 A were divided into three age groups, < 60 years (n = 3), 60 – 70 years (n = 3), and > 70 years (n = 7), and by sex: male (n = 5)

and female (n = 8). (B) The results of Figure 2 B were divided into three age groups, < 60 years (n = 3), 60 – 70 years (n = 2), and > 70 years (n = 1), and by sex: male (n = 2) and female (n = 4). Mean with SD is shown and Mann-Whitney U tests were performed ($p \leqslant 0.05$ (*)).

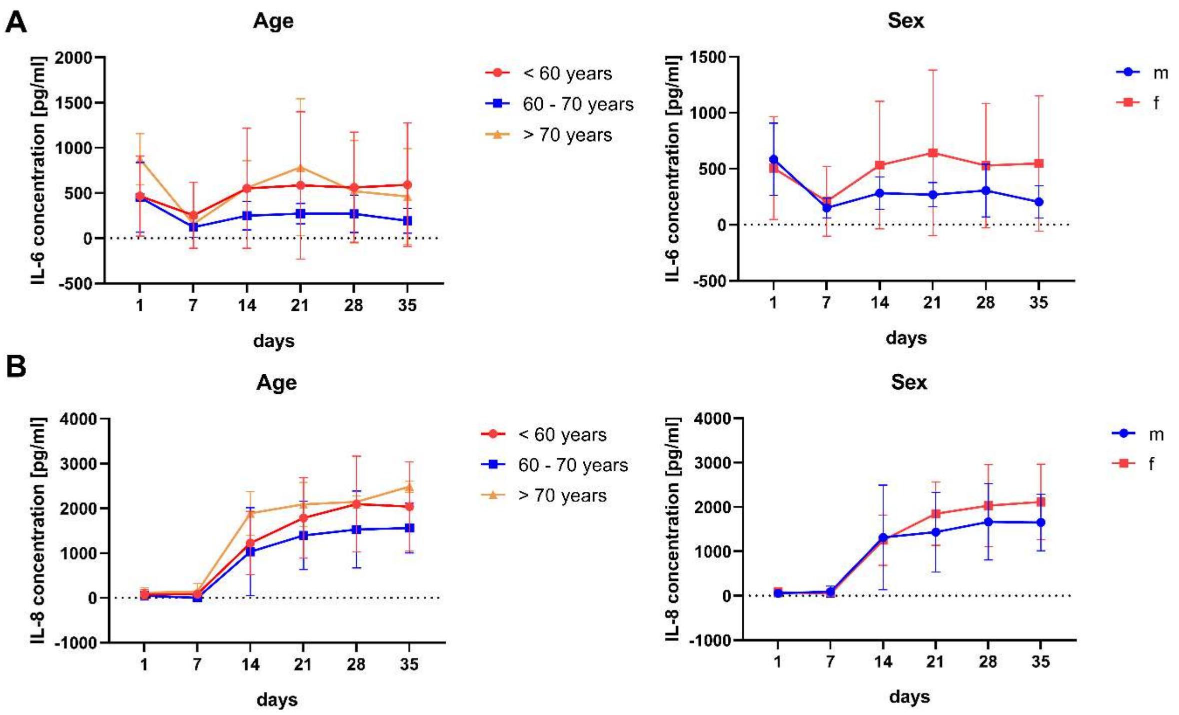

Supplementary Figure S3: Influence of donor age and sex on IL-6 and IL-8 secretion of osteoblast-like cells *in vitro*. The results of (A) IL-6 secretion in Fig. 2 D and (B) IL-8 secretion in Fig. 2 E were divided into three age groups, < 60 years (n = 5), 60 – 70 years (n = 4), and > 70 years (n = 2), and by sex: male (n = 4) and female (n = 7).

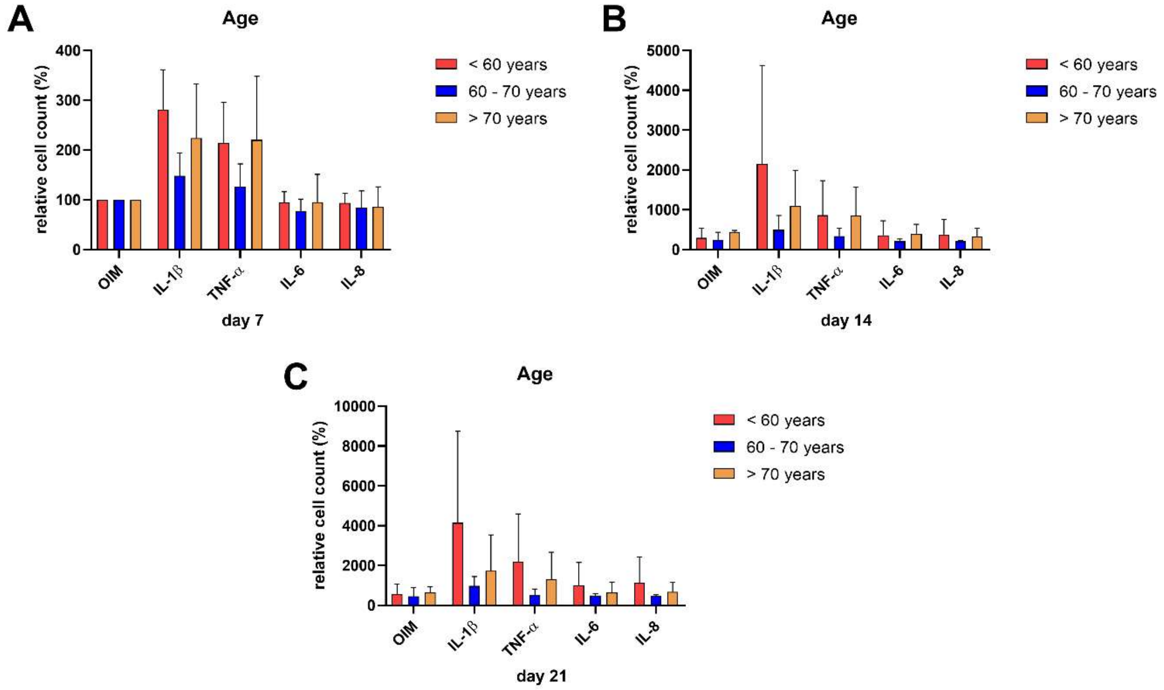

Supplementary Figure S4: Influence of donor age on the cell count during osteogenesis with and without cytokine treatment of osteoblast-like cells *in vitro*. The results of Fig. 5 A-C were divided into three age groups, < 60 years (n = 2), 60 – 70 years (n = 3), and > 70 years (n = 3).
